# New Insights Into the Relationship Between Drought and Mental Health Emerging From the Australian Rural Mental Health Study

**DOI:** 10.3389/fpsyt.2021.719786

**Published:** 2021-09-01

**Authors:** Tuyen T. Luong, Tonelle Handley, Emma K. Austin, Anthony S. Kiem, Jane L. Rich, Brian Kelly

**Affiliations:** ^1^Centre for Resources Health and Safety, University of Newcastle, Callaghan, NSW, Australia; ^2^Centre for Water, Climate, and Land, University of Newcastle, Callaghan, NSW, Australia; ^3^School of Medicine and Public Health, University of Newcastle, Callaghan, NSW, Australia; ^4^Centre for Brain and Mental Health Research, University of Newcastle, Callaghan, NSW, Australia; ^5^Centre for Rural and Remote Mental Health, University of Newcastle, Orange, NSW, Australia

**Keywords:** mental health, drought, resilience, psychological distress, ARMHS

## Abstract

While it is recognized that drought affects mental health, few population-based longitudinal studies quantify this relationship. In this study, we investigate the effects of drought on mental health in a rural population, and how these effects change with continued exposure to drought conditions. Using a panel dataset consisting of 6,519 observations from the Australian Rural Mental Health Study, we found a non-linear (inverted U-shape) relationship between drought exposure and mental health. Specifically, people experienced an increase of psychological distress for the first 2.5–3 years of drought, after which time this distress dissipates. These effects were maintained after controlling for demographic, social, and environmental factors. We also found that while psychological distress decreases in the later stages of drought, this does not necessarily mean people have good mental health because, for example, factors such as life satisfaction decreased as drought persisted. This is important as it highlights the need for sustained support to mitigate the long-term effects of drought on mental health that persist after the drought has apparently finished.

## Introduction

Environmental hazards are important in psychiatry because of their potential risks to mental health and capacity to trigger mental disorders (1). Drought is likely to exacerbate broader risks to mental health by disrupting ecological and socioeconomic systems and amplifying risks to physical health (2). Also, the onset of drought can be stressful when it adversely impacts individual and community economic activities, causes crop and livestock failure; or social isolation and anxiety can occur with the presence of increased workloads, reduced time and resources (2). Understanding the mental health effects of drought is essential to any attempts of drought adaptation and mitigation because mental health and well-being are correlated to adaptive capacity to drought (3).

Drought has been shown to contribute to mental distress (4), suicide, and to creating vulnerabilities for maintaining good health (5–7). Some authors [e.g., (7, 8)] suggest that drought-related financial hardship, lack of water, and migration increase stress and anxiety. While the theoretical relationship between drought and mental health is compelling, the empirical evidence for this is poor.

There is some empirical research exploring the drought-mental health relationship, but this has been limited to using cross-sectional or time-series data, with few studies using longitudinal data. Further, it is difficult to draw conclusions from this small body of work due to the use of different measures for both mental health and drought across different studies. For example, some of the cited studies (9, 10) using general screening mental health measurement (e.g., the Kessler-10 and the SF-36) did not find an association with explicit environmental measures of drought. However, studies using a specific aspect of mental health such as suicide (5, 11, 12), or life satisfaction (13), have found that drought (using environmental measurement) is linearly related to poor mental health outcomes. O' Brien et al. (14) examined drought characteristics as exploratory variables of mental health outcomes and showed that a long drought (20–32 months in an unbroken dry period) is associated with increased psychological distress (measured by the Kessler-10).

On the other hand, there is evidence that drought does not always negatively impact mental health, because of the individual's capacity to respond and to adequately deal with drought. For example, many people in drought-affected communities stay well and have positive mental adjustment to drought (3) or people tend to adapt to their environment (14). Qualitative evidence shows that individuals in farming families utilize psychological coping strategies for drought adaptation such as positive appraisals of their current situation, and optimism (15), or being proactive in risk management and maintaining contact with friends and relatives to help them “get by” during drought (16). With the existence of such evidence, drought therefore might not always be linearly related to poor mental health outcomes.

This suggests that the relationship between drought and mental health is complicated and that many knowledge gaps exist. Some protective factors are assumed to modify an individual's mental response to environmental adversity such as drought (17), but what they are and how they modify this response have not been well-addressed in current research. In addition, the “challenge model” of resilience theory assumes the association between a risk factor and an outcome is non-linear, where continued or repeated risk exposure helps people to adapt to low levels of risk and prepare for more significant risks in the future (18). In other words, people can experience positive outcomes after a threshold level of risk exposure such as drought. Although the non-linear relationship between drought and mental health can be inferred, no empirical evidence has shown this relationship.

We therefore examine the relationship between drought and mental health (both linear and non-linear relationship) with longitudinal data from more than 2,600 respondents in the Australian Rural Mental Health Study (ARMHS). We build on previous research that attempts to quantify the relationship between drought and mental health in two important ways. Firstly, we use two different indices to define drought to see whether our results are sensitive to drought definitions. Secondly, this study also addresses the limitations of most previous approaches by using a longitudinal dataset in which participant outcomes and exposures are observed across time. This enables statistical control for individual characteristics and a more accurate investigation of mental health responses to drought exposure over time. Based on previous research, this paper hypothesizes a linear relationship between drought and mental health. Additionally, it includes exploratory analyses investigating a non-linear relationship between these constructs.

## Data and Methods

### Participants

Our study population comprised participants in the Australian Rural Mental Health Study (ARMHS), a postal survey which aimed to study mental health and its determinants in rural people in New South Wales (NSW), Australia. The Australian Standard Geographic Classification (ASGC) was applied to identify the study population, which included people in four ASGC categories, excluding major cities (inner regional, outer regional, remote, and very remote) (19). The ARMHS data was collected through four waves including a baseline survey and three follow-up surveys administered at 1-, 3-, and 5-years after baseline. The total data collection period was 2007–2013. The design of ARMHS has been reported previously (19). In short, a total of 2,639 participants consented and completed the baseline survey from an initial 9,681 invitations. Although people aged 18–47 years were under-represented in the final sample, the baseline sample was generally representative of the NSW population in comparison to the Australian Bureau of Statistics Census population data (19).

Given the information available in the ARMHS database, we limited the empirical analysis to individuals who lived in NSW throughout the four waves of data collection (i.e., those who did not move to other states after baseline data collection) and provided both the date they completed the survey and their post-code. These variables are essential to measure drought exposure over the time of the study.

### Mental Health Measurement

Mental health was assessed using the Kessler-10 (K10) measure of psychological distress (20) - a prevalent indicator in the study of mental health and well-being (21). The K10 captures psychological distress by asking the frequency of ten symptoms of distress in the last month (e.g., feeling tired out for no good reason, nervous, hopeless, etc.). Each symptom is assessed on a five-point Likert scale corresponding to: “none of the time,” “a little of the time,” “some of the time,” “most of the time,” and “all of the time.” Scores range from 10 to 50 with higher scores indicating greater psychological distress.

Distress is often an acute occurrence, with the K10 measuring symptoms over only a 4-week period. Other aspects of mental health, such as life satisfaction, may be more stable over a longer time period (22). For this reason, we also used the Satisfaction with Life Scale (SWLS) (23) to measure mental health as a sensitivity check. The SWLS asks the level of agreement with five statements about general satisfaction with life (e.g., “In most ways my life is close to my ideal,” “I am satisfied with my life” etc.). Each statement is assessed on a seven-point Likert scale corresponding from “strongly disagree” to “strongly agree.” The scores range from 5 to 35 with higher scores indicating greater life satisfaction.

### Drought Definition

Drought can be defined and measured in many different ways (24, 25). More than 100 indices are used to measure the existence of drought, its duration, and severity for specific areas in given time periods (26). Each drought indicator can also be calculated based on different periods (windows) of antecedent environmental and climate conditions (e.g., rainfall, soil moisture, ground water conditions over the preceding 3, 6, 12, or 60 months) (26).

In this study, we measured drought using the Hutchinson Drought Severity Index (HDSI) (27) and the Standardized Precipitation Evapotranspiration Index (SPEI) (28). The HDSI defines agricultural drought, which refers to dryness in the root zone that leads to a lack of soil moisture supporting vegetation growth, while the SPEI defines meteorological drought, which refers to rainfall deficiencies from average conditions (24). Both meteorological and agricultural indices are used because they are most relevant to the ARMHS population. The methodology used to define drought using HDSI and SPEI is described previously (14, 28). In this paper, the daily precipitation gridded data (~5 km) from the Australian Water Availability Project (AWAP) (29) and daily maximum and minimum temperature gridded data (~5 km) from Australian Bureau of Meteorology (BOM; http://www.bom.gov.au/tempgrids) were used to calculate HDSI and SPEI based on 60-month window. Further details are available from the corresponding author.

### Drought Exposure

The HDSI and SPEI are used to identify the number of drought periods a post-code experienced and the duration of each drought period. Further, the month that individual ARMHS participants completed the survey and the post-code where they lived allowed us to identify the most recent drought period they experienced. We therefore measured drought exposure by calculating the number of years in drought (YID) experienced by ARMHS participants during their most recent drought period.

### Control Variables

Mental health is affected by a large number of different factors. Following the rural mental health literature [e.g., (10, 19, 30, 31)], a set of control variables obtained from ARHMS was included in the empirical analysis. Demographic information included age group, marital status, employment status and perceived financial position (self-reported by participants). A self-rating (five item Likert scale) assessed overall physical health. The List of Threatening Experiences (32) was applied to detect recent adverse life events (in last 12 months) by asking participants whether listed events (e.g., a close person died, arguments or marital difficulties with partner, becoming unemployed, etc.) happened to them. Personality was assessed by the seven-item neuroticism subscale of the Eysenck Personality Questionnaire (33). This subscale asks participants answer seven yes/no questions about personality traits (including being easily hurt, a nervous person, a worrier, being highly strung, suffering from nerves, worrying too long and often guilty) and then was scored by counting the number of “yes” answers. Social interaction was assessed by the Interview Schedule for Social Interaction (ISSI) (34). The ISSI asks participants answer six yes/no questions to assess the availability of interpersonal support (e.g., having someone they can talk to when upset); this is scored by counting the number of “yes” answers. Social networks were assessed by the Berkman Social Network Index (35). This index asks participants about three types of social connections: being currently married; sociability with close friends and relatives (i.e., having ≤ 2 friends and ≤ 2 relatives); or group participation in other community organizations (e.g., charity groups, labor unions, or church groups). Community factors were assessed by the Sense of Community Index (SCI) (36) and the Sense of Place (SOP) tool—a subscale of the Environmental Distress Scale (37). The SCI asks participants to answer 12 true/false statements about the perceived sense of belonging in the local community and is scored by counting the number of “true” answers. The SOP asks respondents to rate their agreement with 10 statements related to feelings about their local area. Each statement is assessed on a five-point Likert scale corresponding from “strongly disagree” to “strongly agree.” The scores range from 5 to 50.

### Data Analysis

To examine the relationship between drought and mental health, we first tested the linear relationship between them by regressing the measure of mental health on the measure of drought exposure (number of years in drought - YID) and a set of control variables (Model 1). Then both drought exposure squared (YID^2^) and drought exposure were used in the model specification to test the potential non-linear relationship between them (Model 2). More detail on the regression equations can be found in Appendix 1.

The main method of data analysis was the population average (PA) or marginal model in the generalized estimating equations (GEEs) framework (38). The PA model describes changes in the average outcome with the covariates across the population while accounting for correlation of repeated measurements in longitudinal data (38, 39). This approach is ideal for our data analysis as the main interest lies in the effect of drought exposure on the mental health response in a population where participant outcomes and exposures are collected at multiple times.

## Results

### Study Population

In total, 2,639 individuals completed the ARMHS baseline survey; our study population comprise 2,607 participants as 32 individuals were excluded (individuals who moved to other states such as Victoria, Queensland after baseline collection and did not provide sufficient information to assess drought exposure and psychological distress). The final sample consists of 6,519 observations across the four waves of data collection. The descriptive statistics of the variables used at baseline and pooled across the four waves are shown in Table 1. The average K10 score is ~15, which is categorized as low psychological distress. The average SWLS is 25, which indicates being slightly satisfied with life. The average level of drought exposure is around 3 continuous years.

**Table 1:** Participants characteristics at baseline and pooled-cross sectional, *n* (%).

**Characteristic**	**Baseline (*n* = 2,607)**	**Pooled-cross sectional (*n* = 6,519)**
Psychological distress (Kessler-10), mean (SD)	14.926 (5.612)	14.170 (5.145)
Life satisfaction (SWLS), mean (SD)	25.703 (6.576)	25.352 (6.484)
Number of years in drought identified by HDSI, mean (SD)	3.017 (1.741)	3.227 (2.057)
Number of years in drought identified by SPEI, mean (SD)	2.760 (1.664)	2.772 (2.071)
Gender (female)	1,547 (59%)	3,945 (61%)
Marital status	**2,595**	**6,486**
Married/De facto	1,952 (75%)	4,928 (76%)
Separated/Divorced/Widowed	433 (17 %)	1,124 (17%)
Never married	210 (8%)	434 (7 %)
Age group	**2,607**	**6,519**
18–34 years	247 (9%)	426 (7%)
35–44 years	372 (14%)	754 (12%)
45–54 years	568 (22%)	1,391 (21%)
55–64 years	697 (27%)	1,840 (28%)
65+ years	723 (28%)	2,108 (33%)
Financial position	**2,274**	**6,154**
Prosperous/very comfortable	354 (16%)	959 (16%)
Reasonably comfortable	1,205 (53%)	3,353 (55%)
Just getting along/poor/very poor	715 (31%)	1,842 (24%)
Physical health	**2,602**	**6,480**
Poor	98 (4%)	240 (4%)
Fair	454 (17%)	1,243 (19%)
Good	925 (36%)	2,504 (38%)
Very good	849 (33%)	1,990 (31%)
Excellent	276 (10%)	503 (98%)
Employment status	**2,588**	**6,482**
Employed	1,460 (56%)	3,504 (54%)
Unemployed	52 (2%)	109 (2%)
Studying/home duties/caring	141 (5%)	300 (5%)
Permanently unable to work	148 (6%)	288 (4%)
Retired	787 (31%)	2,281 (35%)
Social network	**2,027**	**5,771**
Low and medium	914 (45%)	2,485 (43%)
Above medium	1,113 (55%)	3,286 (57%)
Personality	2.006 (1.905)	1.913 (1.387)
Number of stressful life events (0–12), mean (SD)	1.523 (1.566)	1.308 (1.443)
Sense of community	9.053 (2.353)	9.095 (2.363)
Sense of place (10–50), mean (SD)	35.741 (6.393)	35.808 (6.387)
Social interaction (0–6), mean (SD)	5.262 (1.394)	5.280 (1.394)

*SWLS, Satisfaction with Life Scale; SD, Standard Deviation; HDSI, Hutchinson Drought Severity Index; SPEI, Standardized Precipitation Evapotranspiration Index. Bold values indicates the total number of participants with available data for each measure*.

There was some evidence of attrition bias, with participants who only completed baseline reporting a higher K10 score (mean = 15.561, SD = 0.218) than those who completed at least one follow-up survey (mean = 14.619, SD = 0.124) (*t* = 4.028; *p*-value < 0.001).

### Drought and Psychological Distress

#### Linear and Non-linear Relationship

**Figure 1:**
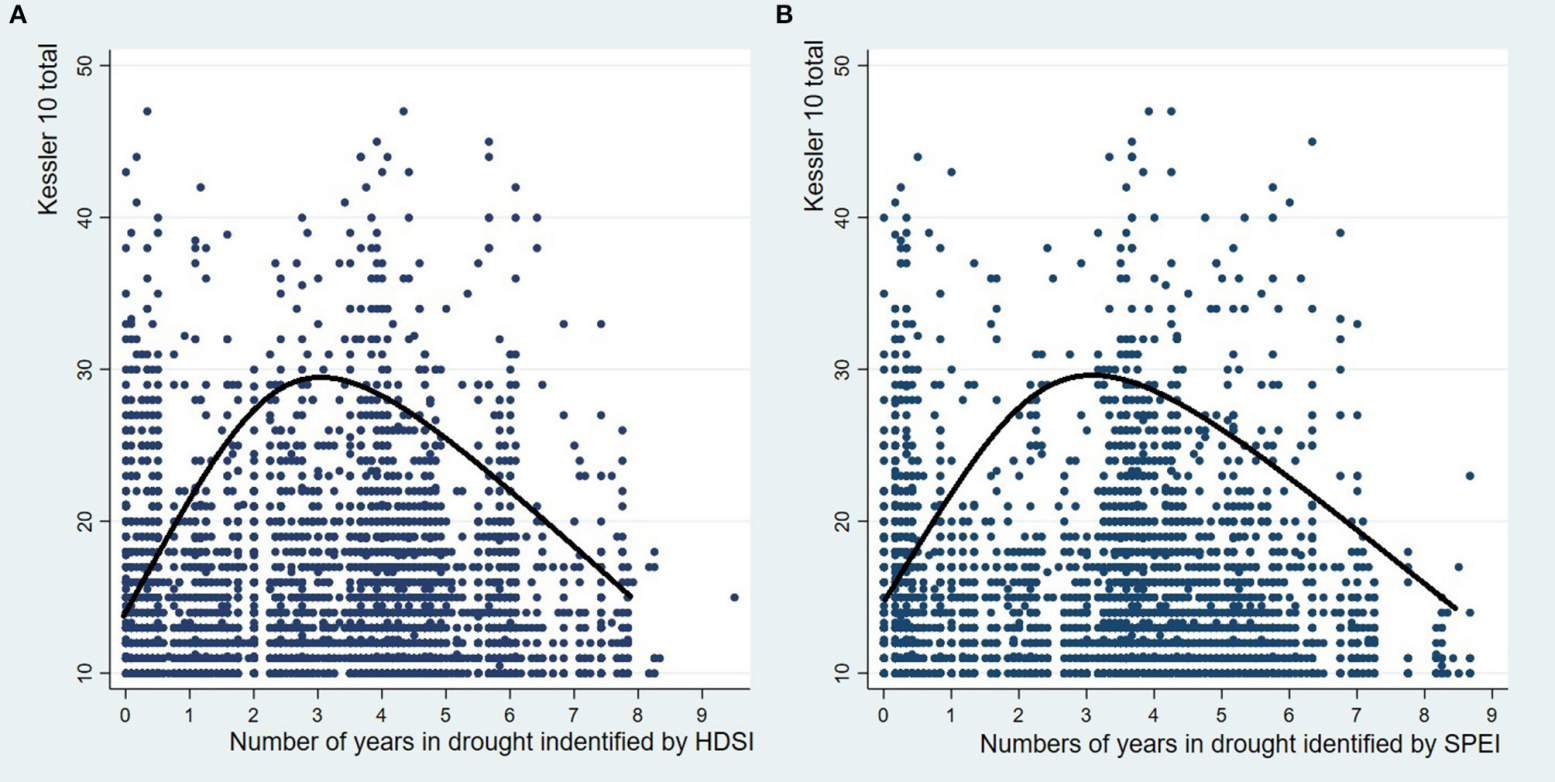
**(A)** The numbers of years in drought identified by the Hutchinson Drought Severity Index (HDSI) and Kessler-10 (K10); **(B)** The numbers of years in drought identified by the Standardized Precipitation Evapotranspiration Index (SPEI) and Kessler-10 (K10).

The regression result from Model 1 did not identify a statistically significant linear relationship between drought exposure and psychological distress (HDSI; *p* = 0.089, and SPEI; *p* = 0.791) (see Supplementary Table 1). However, the estimated coefficients of drought exposure squared (YID^2^) suggest a significant non-linear relationship between drought exposure and psychological distress (HDSI; *p* < 0.001, and SPEI; *p* < 0.001) (See Table 2). Also, the negative coefficients indicated that this relationship follows an inverted U-shape, where drought initially leads to increased psychological distress, but then distress begins to decrease after a threshold level of drought exposure. The turning point of the inverted U-shape (where distress begins to decrease) is found at around 3 years in drought (identified by both HDSI and SPEI). In order to demonstrate the non-linear patterns, a representation of the inverted U-shape is shown in Figure 1.

**Table 2:** Regression results testing the non-linear relationship between drought and mental health.

**Variable**	**HDSI**	**SPEI**
	**coefficient (Standard error)**	**coefficient (Standard error)**
Drought exposure squared (YID^2^)	−0.058*** (0.009)	−0.068*** (0.010)
Drought exposure (YID)	0.351*** (0.072)	0.409*** (0.064)
Gender	Reference group: male	
Female	0.126 (0.121)	0.132 (0.122)
Marital status	Reference group: married	
Separated/divorced/widowed	0.503** (0.193)	0.486** (0.196)
Never married	0.160 (0.264)	0.181 (0.264)
Age group	Reference group: 18–34 years	
35–44 years	−0.100 (0.262)	−0.036 (0.262)
45–54 years	−0.460 (0.265)	−0.398 (0.266)
55–64 years	−1.159*** (0.262)	−1.094*** (0.263)
65+ years	−1.760*** (0.282)	−1.695*** (0.285)
Financial position	Reference group: prosperous/very comfortable	
Reasonably comfortable	0.212 (0.111)	0.229* (0.112)
Just getting along/poor/very poor	0.723*** (0.151)	0.743*** (0.154)
Physical health	Reference group: poor	
Fair	−2.464*** (0.478)	−2.452*** (0.478)
Good	−3.995*** (0.472)	−3.983*** (0.472)
Very good	−4.737*** (0.474)	−4.728*** (0.474)
Excellent	−5.295*** (0.483)	−5.283*** (0.483)
Employment status	Reference group: employed	
Unemployed	0.743 (0.580)	0.667 (0.561)
Studying/home duties/caring	0.281 (0.243)	0.242 (0.240)
Permanently unable to work because of illness	1.528*** (0.433)	1.498*** (0.439)
Retired	0.018 (0.136)	0.035 (0.138)
Social network	Reference group: low/medium	
Above medium	−0.350** (0.113)	−0.342** (0.113)
Personality	0.397*** (0.039)	0.414*** (0.040)
Number of stressful life events	0.517*** (0.043)	0.512*** (0.043)
Sense of community	−0.102*** (0.026)	−0.100*** (0.026)
Social interaction	−0.415*** (0.051)	−0.414*** (0.051)
Sense of place	−0.007 (0.009)	−0.007*** (0.009)
Wald chi-square (degree of freedom)	1,181 (25)***	1,228 (25)***
Number of observation	5,438	5,438
Number of individuals	2,196	2,196
Turning point (years)	3.023	2.998

*Significant at 0.05 level; **at 0.01 level; ***at 0.001 level; Robust standard errors are in parentheses. All regressions include a constant; HDSI, Hutchinson Drought Severity Index; SPEI, Standardized Precipitation Evapotranspiration Index.

#### Time Since Drought

After the non-linear relationship between drought and psychological distress was confirmed, a further analysis (that included the numbers of years since the drought occurred) was conducted. The results in Supplementary Table 2 indicate that controlling for time since drought exposure does not eliminate the inverted U-shape relationship between drought exposure and psychological distress. In other words, the non-linear relationship between them is robust in spite of controlling for a new variable related to drought. The turning point of this inverted U-shape was found at around 2.5 and 3 years in drought (identified by HDSI and SPEI, respectively).

### Drought and Satisfaction With Life

The results in Table 3 show that drought exposure, identified by HDSI, is negatively related to life satisfaction (the coefficient is −0.083, *p*-value = 0.045). This suggests that individuals who experienced a higher number of years in drought tend to feel less satisfied with life. Holding all other variables constant, a 1-year increase in drought exposure reduces satisfaction with life by 0.083 points (on the 5–35 scale). Despite of the small effect, this empirical result can be interpreted as supporting the negative effects of drought on mental health. When using SPEI to define drought, the effect of drought exposure on life satisfaction was statistically non-significant. In addition, there was no significant non-linear relationship between drought exposure and life satisfaction when using either HDSI or SPEI to measure drought exposure (See Supplementary Table 3).

**Table 3:** Regression results testing the linear relationship between drought and life satisfaction.

**Variable**	**HDSI**	**SPEI**
	**Coefficient (Standard error)**	**Coefficient (Standard error)**
Drought exposure	−0.083* (0.041)	−0.012 (0.041)
Gender	Reference group: male	
Female	0.019 (0.197)	0.018 (0.197)
Marital status	Reference group: married	
Separated/divorced/widowed	−1.902*** (0.259)	−1.887*** (0.259)
Never married	−1.933*** (0.391)	−1.912*** (0.391)
Age group	Reference group: 18–34 years	
35–44 years	−1.281** (0.424)	−1.276** (0.424)
45–54 years	−1.911*** (0.413)	−1.897*** (0.413)
55–64 years	−1.081** (0.415)	−1.075* (0.416)
65+ years	−0.753 (0.463)	−0.755 (0.463)
Financial position	Reference group: prosperous/very comfortable	
Reasonably comfortable	−1.486*** (0.218)	−1.477*** (0.219)
Just getting along/poor/very poor	−3.615*** (0.260)	−3.602*** (0.260)
Physical health	Reference group: poor	
Fair	1.658*** (0.455)	1.625*** (0.455)
Good	2.977*** (0.449)	2.950*** (0.449)
Very good	3.837*** (0.459)	3.812*** (0.459)
Excellent	4.817*** (0.513)	4.797*** (0.514)
Employment status	Reference group: employed	
Unemployed	−0.667 (0.594)	−0.684 (0.594)
Studying/home duties/caring	0.054 (0.366)	0.059 (0.367)
Permanently unable to work because of illness	−1.101** (0.411)	−1.092** (0.411)
Retired	0.059 (0.249)	0.067 (0.249)
Social network	Reference group: low/medium	
Above median	0.570** (0.188)	0.577** (0.188)
Personality	−0.319*** (0.051)	−0.318*** (0.051)
Number of stressful life events	−0.523*** (0.055)	−0.522*** (0.055)
Sense of community	0.199*** (0.040)	0.199*** (0.041)
Social interaction	0.645*** (0.061)	0.647*** (0.061)
Sense of place	0.239*** (0.015)	0.238*** (0.015)
Wald chi- square (degree of freedom)	2,223 (24)***	2,219 (24)***
Number of observation	3,948	3,948
Number of individuals	2,096	2,096

*Significant at 0.05 level; **at 0.01 level; ***at 0.001 level; Robust standard errors are in parentheses. All regressions include a constant; HDSI, Hutchinson Drought Severity Index; SPEI, Standardized Precipitation Evapotranspiration Index.

## Discussion

This paper is a first step toward understanding the relationship between drought exposure and mental health through modeling. By using longitudinal analyses, we provide evidence that the psychological response to drought changes with different levels of drought exposure and follows an inverted U-shape pattern. This finding is consistent across two definitions of drought, where drought exposure initially led to increased psychological distress, and subsequently, after a threshold level of drought exposure (around 2.5–3 years), distress begins to decrease.

The increase of distress in the first years of drought suggests that people tend to be more distressed when experiencing more time in drought. However, after a period of time in drought, the distress begins decreasing. This is in line with O' Brien et al. (14) who noted that people tend to adapt to their environment and to develop mental resilience in the face of severe or prolonged stressors. Moreover, while people ultimately are likely to adjust to drought (i.e., their distress begins decrease), it takes about 3 years for this to occur, and during which time distress consistently increases. This might be because it takes time (e.g., months or years) after droughts have started to be able to see or identify drought (24), and drought impacts also develop gradually and can persist for years (24). Therefore, the first years of drought could bring many uncertainties as well as potential risks to factors such as financial security, leading to persisting distress. This finding also supports the need to improve drought predictions/projections in terms of starting time, its duration, magnitude and spatial extent to create opportunities for proactive mitigation or adaptation of communities and stakeholders (24).

In our study, distress eventually decreased as drought exposure continued. However, a decrease in distress as drought conditions continue does not reflect a return to positive overall mental health. We found that the high level of drought exposure was associated with a decrease in general satisfaction with life over time. This is in line with Carroll et al. (13), who indicates that the longer time spent in drought increases the likelihood of a decline in life satisfaction. In other words, when people are no longer distressed about drought, this does not necessarily mean their overall mental health is restored. This is supported by the finding/suggestion that it is not the magnitude of a relative dry period that matters for mental health, but how long it persists (14). This is an important finding because reduction in distress after a few years of drought exposure could be mistaken for an indication that drought assistance interventions have been successful and can be relaxed; when in fact this assistance should be continued, and possibly increased, until the drought has broken and its long-term impacts have diminished. This also suggests that continuous support would be advantageous for better life satisfaction outcomes for people in drought-affected areas. One of the ways to support people's well-being might be through place attachment, which is defined as the emotional bond with environmental settings (40). This is because place attachment was found to enhance life satisfaction and to contribute to greater happiness (41, 42). Also, the measure of improving mental health as an outcome of such programs or as a criteria of program valuation would be helpful, especially if it does not rely on only one indicator such as psychological distress.

### Limitations and Future Directions

Although this study makes clear contributions to the existing literature, it has a number of limitations. The bias from self-reported data related to determinants of mental health is unavoidable in our study. In term of attrition bias, the fact that distress reduced over time could not only suggest the capacity to adapt to drought, but also mean that people who are very distressed (have a high K10 score) at baseline stopped responding to follow-up surveys, reducing the overall mean of the sample. However, the difference in mean baseline K10 between people who dropped out and remained in the study is relatively small (<1 point on the K10 scale). Therefore, our analysis would introduce limited bias from participant dropouts. Moreover, the calculation of meteorological conditions (temperatures and precipitation) for each post-code as a proxy for drought exposure of all participants in that area would have introduced some measurement error into the analysis, especially as, in Australia, some post-codes can be large. However, with the approach of calculating the average of temperature and rainfall for post-codes based on grid cell's data record, our analysis would lessen the potential impact of any errors.

Although protective factors (that help to moderate the negative effects of drought on mental health and/or to promote psychological resilience among rural people) are not directly investigated in this study, our analysis suggested the existence of such factors in the drought-mental health relationship. Having a better understanding of what helps people “get by” during periods of drought, while others experience negative outcomes, is fundamental in the process of identifying clear-cut and well-defined goals of interventions and adaptation options. Future research should aim to identify and understand these factors.

Also, further research is required to clarify and quantify pathways (mechanism) of how drought affects mental health outcomes. For long-term policies in mitigating the impacts of drought on mental health, it is essential to build a positive adaptation to drought on the basis of improving adaptive capacity and mental resilience and/or to integrate mental health care in drought adaptation strategies.

The importance of environmental adversity such as drought was demonstrated by significant influences on mental health outcomes. In addition, it is possible that other environmental events such as severe storms, floods or heatwaves also affect communities. Further studies will be necessary to explore the effects of these events because they are complicated and may combine and cumulatively affect mental health outcomes.

## Conclusion

It is well-established that the relationship between drought and mental health is complex. However, little is known about (i) how drought duration affects (associates with) mental health (especially when the drought lasts for years) and (ii) how long poor mental health persists after a drought. In this paper, we took a novel approach that allowed us to explore these questions. We found that the association between drought exposure and psychological distress is non-linear (inverted U-shape). More specifically, people experienced an increase in psychological distress for the first 2.5–3 years of drought, after which time distress dissipates. To our knowledge, this is the first paper that exhibits an inverted U-shape for the drought-psychological distress relationship with both theoretical and empirical explanation. This finding suggests the existence of factors that help to moderate the negative effects of drought on mental health or to promote psychological resilience to drought. Further research is required to identify and understand such factors.

We also found that a decrease in distress after a threshold of drought exposure does not mean people have positive overall mental health. This was evidenced by the fact that the increased level of drought exposure was associated with decreased life satisfaction over time. This is vital as it emphasizes the need for sustained support to mitigate the long-term effects of drought on mental health that persist after the drought has apparently finished. It also highlights the importance of using different measures of mental health in both research and practices related to drought and mental health.

## Data Availability Statement

The data analyzed in this study is subject to the following licenses/restrictions: The dataset from the Australian Rural Mental Health Study (ARMHS) is restricted excepting for investigators (Chief Investigator and co-investigators) and research students who are approved by Human Research Ethics Committee. Requests to access these datasets should be directed to Prof. Brian Kelly (Chief Investigator of ARMHS). Email: brian.kelly@newcastle.edu.au.

## Ethics Statement

The studies involving human participants were reviewed and approved by Human Research Ethics Administration (The University of Newcastle Callaghan NSW 2308). The patients/participants provided their written informed consent to participate in this study.

## Author Contributions

TH, AK, EA, JR, and TL involved in data preparation, data analysis, and interpretation. TL drafted and revised the manuscript. All authors conceived, designed the study, reviewed, and approved the final version of the manuscript.

## Conflict of Interest

The authors declare that the research was conducted in the absence of any commercial or financial relationships that could be construed as a potential conflict of interest.

## Publisher's Note

All claims expressed in this article are solely those of the authors and do not necessarily represent those of their affiliated organizations, or those of the publisher, the editors and the reviewers. Any product that may be evaluated in this article, or claim that may be made by its manufacturer, is not guaranteed or endorsed by the publisher.
