# Erratum: New insights into the relationship between drought and mental health emerging from the Australian rural mental health study

**DOI:** 10.3389/fonc.2023.1145296

**Published:** 2023-02-14

**Authors:** Frontiers Production Office

**Affiliations:** Frontiers Media SA, Lausanne, Switzerland

**Keywords:** mental health, drought, resilience, psychological distress, ARMHS

Due to a production error, the word “measured” was removed from a sentence.

A correction has been made to the section **Data and Methods,** subsection **Drought Definition**, Paragraph 2, first sentence:

“In this study, we measured drought using the Hutchinson Drought Severity Index (HDSI) (27) and the Standardized Precipitation Evapotranspiration Index (SPEI) (28).”

The publisher apologizes for this mistake. The original version of this article has been updated.

