# Supplementary material for: New Insights Into the Relationship Between Drought and Mental Health Emerging From the Australian Rural Mental Health Study
Source: Front Psychiatry. 2021 Sep 1;12:719786. doi: 10.3389/fpsyt.2021.719786 (PMC8440818; doi:10.3389/fpsyt.2021.719786)
Supplement: Supplementary file 1 [file Data_Sheet_1.docx]

***Supplementary Material***

**APPENDIX 1: Empirical models**

The linear and non-linear relationship between mental health outcome and predictor variables including drought exposure can be described by Equation 1 and Equation 2, respectively as follows.

${MH}_{it}=\beta_{0}+\beta_{1}{YID}_{it} +\sum_{j=2}^{j} \beta_{j}X_{ijt}+\varepsilon_{it}$ (1)

${MH}_{it}=\beta_{0}+\beta_{1}{YID}_{it}+\beta_{2}{{YID}^{2}}_{it} +\sum_{j=3}^{j} \beta_{j}X_{ijt}+\varepsilon_{it}$ (2)

Where:

- ${MH}_{it}$ is mental health of individual i at time t.
- $\beta_{0}$is the intercept.
- ${YID}_{it}$ is the number of years in the most recent drought (drought exposure) of individual i at time t.
- $\beta_{1}$ and $\beta_{2}$ are the regression coefficients of YID_it_ and YID^2^_it_.
- j is the number of independent variables
- $X_{ijt}$ is the independent variable (control variable) j (such as age group, gender….) for individual i at time t.
- $\beta_{j}$is the regression coefficient for independent variable j.
- $\varepsilon_{it}$ is error terms for individual i at time t

**APPENDIX 2: SUPPLEMENTARY TABLE**

Table S1: Regression results of testing the linear relationship between drought and mental health

| **Variable** | **HDSI**  **Coefficient (Standard error)** | **SPEI**  **Coefficient (Standard error)** |
| --- | --- | --- |
| Drought exposure | -0.042 (0.025) | 0.006 (0.024) |
| Gender | *Reference group: Male* | |
| Female | 0.108 (0.122) | 0.111 (0.122) |
| Marital status | *Reference group: Married* | |
| *Separated/Divorced/Widowed* | 0.507** (0.195) | 0.511** (0.194) |
| *Never married* | 0.180 (0.267) | 0.192 (0.267) |
| Age group | *Reference group: 18 – 34 years* | |
| *35-44 years* | -0.123 (0.266) | -0.113 (0.265) |
| *45-54 years* | -0.483 (0.269) | -0.465 (0.268) |
| *55-64 years* | -1.201*** (0.264) | -1.186*** (0.265) |
| *65+ years* | -1.794*** (0.285) | -1.784*** (0.285) |
| Financial position | *Reference group: Prosperous/Very comfortable* | |
| *Reasonably comfortable* | 0.247* (0.112) | 0.251* (0.112) |
| *Just getting along/ Poor/Very poor* | 0.789*** (0.153) | 0.792*** (0.152) |
| Physical health | *Reference group: Poor* | |
| *Fair* | -2.437*** (0.476) | -2.457*** (0.479) |
| *Good* | -3.939*** (0.470) | -3.959*** (0.473) |
| *Very good* | -4.686*** (0.472) | -4.708*** (0.474) |
| *Excellent* | -5.239*** (0.481) | -5.258*** (0.484) |
| Employment status | *Reference group: Employed* | |
| *Unemployed* | 0.730 (0.567) | 0.716 (0.560) |
| *Studying/Home Duties/Caring* | 0.289 (0.244) | 0.287 (0.243) |
| *Permanently unable to work because of illness* | 1.557*** (0.436) | 1.560*** (0.436) |
| *Retired* | 0.007 (0.138) | 0.011 (0.138) |
| Social network | *Reference group: Low or medium* | |
| *Above medium* | -0.332** (0.114) | -0.329** (0.114) |
| Personality | 0.391*** (0.039) | 0.391*** (0.039) |
| Number of stressful life events | 0.519*** (0.043) | 0.519*** (0.043) |
| Sense of community | -0.104*** (0.026) | -0.104*** (0.026) |
| Social interaction | -0.415*** (0.051) | -0.414*** (0.051) |
| Sense of place | -0.007 (0.009) | -0.007 (0.009) |
| Wald chi-square (degree of freedom) | 1139 (24)**** | 1135 (24)*** |
| Number of observation | 5438 | 5438 |
| Number of individuals | 2196 | 2196 |

(*Significant at 0.05 level; ** at 0.01 level; *** at 0.001 level; Robust standard errors are in parentheses. All regressions include a constant; HDSI denotes the Hutchinson Drought Severity Index; SPEI denotes the Standardized Precipitation Evapotranspiration Index)

Table S2: Further analysis that included time since drought

| **Variable** | **HDSI**  **Coefficient (Standard error)** | **SPEI**  **Coefficient (Standard error)** |
| --- | --- | --- |
| Drought exposure squared (YID^2^) | -0.040*** (0.010) | -0.063*** (0.010) |
| Drought exposure (YID) | 0.198** (0.083) | 0.361*** (0.065) |
| Time since drought | -0.159*** (0.022) | -0.157*** (0.022) |
| Gender | *Reference group: Male* | |
| Female | 0.169 (0.126) | 0.145 (0.124) |
| Marital status | *Reference group: Married* | |
| *Separated/Divorced/Widowed* | 0.520** (0.207) | 0.514** (0.201) |
| *Never married* | 0.163 (0.274) | 0.196 (0.268) |
| Age group | *Reference group: 18-34 years* | |
| *35-44 years* | -0.018 (0.274) | 0.048 (0.261) |
| *45-54 years* | -0.345 (0.278) | -0.319 (0.267) |
| *55-64 years* | -1.000*** (0.275) | -0.986*** (0.266) |
| *65+ years* | -1.591*** (0.298) | -1.523*** (0.290) |
| Financial position | *Reference group: Prosperous/Very comfortable* | |
| *Reasonably comfortable* | 0.218 (0.115) | 0.181 (0.113) |
| *Just getting along/ Poor/Very poor* | 0.732*** (0.160) | 0.713*** (0.158) |
| Physical health | *Reference group: Poor* | |
| *Fair* | -2.515*** (0.503) | -2.314*** (0.483) |
| *Good* | -3.986*** (0.496) | -3.865*** (0.478) |
| *Very good* | -4.770*** (0.497) | -4.638*** (0.479) |
| *Excellent* | -5.426*** (0.507) | -5.208*** (0.489) |
| Employment status | *Reference group: Employed* | |
| *Unemployed* | 0.757 (0.589) | 0.721 (0.571) |
| *Studying/Home Duties/Caring* | 0.266 (0.250) | 0.266 (0.244) |
| *Permanently unable to work because of illness* | 1.488*** (0.462) | 1.493*** (0.450) |
| *Retired* | 0.057 (0.139) | 0.084 (0.138) |
| Social network | *Reference group: Low/ medium* | |
| *Above median* | -0.335** (0.117) | -0.364** (0.115) |
| Personality | 0.394*** (0.041) | 0.396*** (0.041) |
| Number of stressful life events | 0.512*** (0.045) | 0.505*** (0.043) |
| Sense of community | -0.113*** (0.027) | -0.099*** (0.026) |
| Social interaction | -0.401*** (0.052) | -0.412*** (0.052) |
| Sense of place | -0.011 (0.009) | -0.010 (0.009) |
| Number of observation | 5102 | 5303 |
| Number of individuals | 2065 | 2145 |
| Turning point (years) | 2.475 | 2.865 |

(*Significant at 0.05 level; ** at 0.01 level; *** at 0.001 level; Robust standard errors are in parentheses. All regressions include a constant; HDSI denotes the Hutchinson Drought Severity Index; SPEI denotes the Standardized Precipitation Evapotranspiration Index).

Table S3: Regression results testing the non-linear relationship between drought and life satisfaction

| **Variable** | **HDSI**  **Coefficient (Standard error)** | **SPEI**  **Coefficient (Standard error)** |
| --- | --- | --- |
| Drought exposure squared (YID^2^) | -0.029 (0.017) | -0.022 (0.017) |
| Drought exposure (YID) | 0.123 (0.127) | 0.119 (0.107) |
| Gender | *Reference group: Male* | |
| *Female* | 0.028 (0.197) | 0.024 (0.197) |
| Marital status | *Reference group: Married* | |
| *Separated/Divorced/Widowed* | -1.903*** (0.259) | -1.896*** (0.259) |
| *Never married* | -1.940*** (0.391) | -1.916*** (0.391) |
| Age group | *Reference group:* | |
| *35-44 years* | -1.269** (0.424) | -1.252** (0.424) |
| *45-54 years* | -1.890*** (0.413) | -1.872*** (0.414) |
| *55-64 years* | -1.060* (0.415) | -1.047* (0.416) |
| *65+ years* | -0.736 (0.463) | -0.724 (0.464) |
| Financial position | *Reference group: Prosperous/Very comfortable* | |
| *Reasonably comfortable* | -1.503*** (0.219) | -1.484*** (0.219) |
| *Just getting along/ Poor/Very poor* | -3.650*** (0.260) | -3.618*** (0.260) |
| Physical health | *Reference group: Poor* | |
| *Fair* | 1.639*** (0.455) | 1.617*** (0.455) |
| *Good* | 2.944*** (0.449) | 2.932*** (0.449) |
| *Very good* | 3.803*** (0.459) | 3.792*** (0.459) |
| *Excellent* | 4.785*** (0.514) | 4.774*** (0.514) |
| Employment status | *Reference group: Employed* | |
| *Unemployed* | -0.688 (0.594) | -0.697 (0.594) |
| *Studying/Home Duties/Caring* | 0.039 (0.367) | 0.043 (0.367) |
| *Permanently unable to work because of illness* | -1.106** (0.411) | -1.110** (0.411) |
| *Retired* | 0.067 (0.249) | 0.069 (0.249) |
| Social network | *Reference group: Low/medium* | |
| *Above medium* | 0.560** (0.188) | 0.572** (0.188) |
| Personality | -0.317*** (0.051) | -0.311*** (0.051) |
| Number of stressful life events | -0.524*** (0.055) | -0.524*** (0.055) |
| Sense of community | 0.199*** (0.040) | 0.199*** (0.041) |
| Social interaction | 0.645*** (0.061) | 0.647*** (0.061) |
| Sense of place | 0.239*** (0.015) | 0.239*** (0.015) |
| Wald chi-square | 2228.280 | 2219.660 |
| Number of observation | 3948 | 3948 |
| Number of individuals | 2096 | 2096 |
| Turning point (years) | Not available | Not available |

(*Significant at 0.05 level; ** at 0.01 level; *** at 0.001 level; Robust standard errors are in parentheses. All regressions include a constant; HDSI denotes the Hutchinson Drought Severity Index; SPEI denotes the Standardized Precipitation Evapotranspiration Index)
